# Deep Sequencing of MYC DNA-Binding Sites in Burkitt Lymphoma

**DOI:** 10.1371/journal.pone.0026837

**Published:** 2011-11-10

**Authors:** Volkhard Seitz, Peter Butzhammer, Burkhard Hirsch, Jochen Hecht, Ines Gütgemann, Anke Ehlers, Dido Lenze, Elisabeth Oker, Anke Sommerfeld, Edda von der Wall, Christoph König, Christian Zinser, Rainer Spang, Michael Hummel

**Affiliations:** 1 Institute of Pathology, Charité - University Medicine, Campus Benjamin Franklin, Berlin, Germany; 2 Institute for Functional Genomics, University of Regensburg, Regensburg, Germany; 3 Berlin-Brandenburg Center for Regenerative Therapies (BCRT), Charité - University Medicine, Berlin, Germany; 4 Department of Pathology, University Hospital of Bonn, Bonn, Germany; 5 imaGenes GmbH, Berlin, Germany; 6 Genomatix Software GmbH, Munich, Germany; University of Nebraska – Lincoln, United States of America

## Abstract

**Background:**

MYC is a key transcription factor involved in central cellular processes such as regulation of the cell cycle, histone acetylation and ribosomal biogenesis. It is overexpressed in the majority of human tumors including aggressive B-cell lymphoma. Especially Burkitt lymphoma (BL) is a highlight example for MYC overexpression due to a chromosomal translocation involving the *c-MYC* gene. However, no genome-wide analysis of MYC-binding sites by chromatin immunoprecipitation (ChIP) followed by next generation sequencing (ChIP-Seq) has been conducted in BL so far.

**Methodology/Principal Findings:**

ChIP-Seq was performed on 5 BL cell lines with a MYC-specific antibody giving rise to 7,054 MYC-binding sites after bioinformatics analysis of a total of approx. 19 million sequence reads. In line with previous findings, binding sites accumulate in gene sets known to be involved in the cell cycle, ribosomal biogenesis, histone acetyltransferase and methyltransferase complexes demonstrating a regulatory role of MYC in these processes. Unexpectedly, MYC-binding sites also accumulate in many B-cell relevant genes. To assess the functional consequences of MYC binding, the ChIP-Seq data were supplemented with siRNA- mediated knock-downs of MYC in BL cell lines followed by gene expression profiling. Interestingly, amongst others, genes involved in the B-cell function were up-regulated in response to MYC silencing.

**Conclusion/Significance:**

The 7,054 MYC-binding sites identified by our ChIP-Seq approach greatly extend the knowledge regarding MYC binding in BL and shed further light on the enormous complexity of the MYC regulatory network. Especially our observations that (i) many B-cell relevant genes are targeted by MYC and (ii) that MYC down-regulation leads to an up-regulation of B-cell genes highlight an interesting aspect of BL biology.

## Introduction

MYC is a transcription factor encoded by the *c-MYC* gene (thereafter termed *MYC*) which regulates an estimated 15% of genes in the human genome [1]. MYC is a helix-loop-helix leucine zipper transcription factor which dimerizes with MYC-associated factor X (MAX) to bind to the DNA consensus motive (CACGTG), known as Enhancer Box (E-box) [1]. Together, MYC and MAX coordinately regulate the transcription of distinct genes involved in cell cycle progression, differentiation, apoptosis, transformation and genomic instability [2], [3]. Elevated MYC expression can be found in up to 70% of human tumors, and suppression of MYC is thought to lead to tumor regression [4].

One of the mechanisms of MYC-driven gene activation is the increase of the local histone acetylation at promoter sites. Once bound to its target promoter, MYC can interact with histone modifiers, such as histone acetylases (HATs), GCN5/PCAF, P300/CBP, TIP60 or HAT-associated proteins (e.g. TRRAP), resulting in local hyper-acetylation of histones [5], [6], [7]. Furthermore, MYC can promote transcription by stimulating the RNA polymerase II [8], [9].

MYC can also inhibit transcription of genes (e.g. *P15*, *P21*, *P27*) by blocking the respective activation factors such as SMAD, YY-1, SP1, MIZ-1, TFII-I and NF-Y [10]. Furthermore, MYC may also repress transcription by recruitment of the DNA methyltransferase co-repressor DMNT3A [11]. DNA methylation is an important epigenetic modification and associated with transcriptional silencing. MYC itself is also part of a complex regulatory network, were MAD family members MAD1, MAD3, MAD4, MXI1, MNT and MGA function in part as MYC antagonists [12]. Within this network, MYC has the ability to activate and repress gene transcription [13], [14]. The control of micro RNAs (miRNAs) by MYC, which can influence the cell cycle, apoptosis, metabolism and tumor metastasis, was described only recently [15].

MYC was first discovered in Burkitt lymphoma (BL), which harbors a chromosomal translocation of *MYC*. This translocation leads to a fusion of *MYC* to one of the three immunoglobulin (*Ig*) loci [16], [17]. According to the new WHO classification, three variants of BL are recognized, namely endemic BL (eBL), sporadic BL (sBL) and immunodeficiency-associated BL (iBL), which are related very closely to each other according to our recent findings [18], [19]. Most likely, BL cells are derived from germinal center experienced B-cells. The expression of germinal center markers (e.g. BCL6, CD10) and the finding of somatic hypermutations in their Ig genes serve as evidence [18], [20], [21], [22].

MYC-binding sites were previously analyzed by ChIP-on-chip (Chromatin immunoprecipitation in combination with promoter tiling arrays) and by ChIP-PET (Paired end sequencing of the precipitated DNA fragments) analysis, in a single BL cell line and one MYC-inducible lymphoblastoid cell line (P493-6), respectively, latter serving as BL model [23], [24]. Through recent advances of high throughput sequencing, ChIP combined with massively parallel DNA sequencing (ChIP-Seq) became a new option to identify genome-wide binding sites of DNA-associated proteins [25].

The primary goal of the present study thus was the generation of a genome-wide map of MYC-binding sites in BL genomes. To this end, we carried out ChIP in 5 human BL cell lines, employing a MYC-specific antibody followed by next generation sequencing of the precipitated DNA fragments. We identified 7,054 MYC-binding sites and used the associated genes to detect functionally relevant gene sets. In addition we performed MYC knock-down experiments in BL cell lines followed by gene expression profiling. Similar functional groups of genes were found by ChIP-Seq and by MYC silencing. Of special interest was, however, our finding that – in addition to already established MYC target genes – genes important for the function and immunogenicity of B-cells are targeted by MYC binding and are up-regulated by MYC inhibition.

## Methods

### Cell culture

Five human Burkitt lymphoma (BL) cell lines (Raji, CA46, Blue1, BL41, Ramos) were purchased from the German Collection of Microorganisms and Cell Cultures (DSMZ, Braunschweig, Germany) and cultured with 5% CO_2_ in RPMI 1640 (PAA, Pasching, Austria) supplemented with 10% Sera Plus (PAN Biotech, Aidenbach, Germany) at 37°C.

### ChIP experiments

A polyclonal MYC antibody (N-262: LOT E2308) Santa Cruz, CA, USA) was employed for ChIP. The specificity and suitability of this antibody for ChIP has already been shown by previously published work [24]. The ChIP experiments were carried out according to the protocol developed by the group of R.A. Young with minor modifications and with input DNA as control [26]. ChIP was performed in 3 separate volumes of 1 ml with 10 µg MYC antibody (100 µl beads) for each cell line.

Successful enrichment (threshold >20-fold) of MYC-bound DNA fragments was determined by real-time DNA-PCR with primers for *NPM1*, an already well defined MYC target gene [24]. Real-time DNA-PCR was performed with SYBR Green PCR-Master Mix on a 7900HT Fast Real-Time PCR cycler (both Applied Biosystems, Foster City, CA, USA) using the PCR parameters recommended by the manufacturer.

Relative quantification of real-time DNA-PCR results was calculated using the comparative ΔΔCT method [27]. For normalization of real-time DNA-PCR, primers annealing to the 3′- end of the *PRAME* gene were employed, with the exception of the Ramos cell line where the *PRAME* 3′- end could not be amplified, and therefore primers annealing to the *ACTIN* gene were used for normalization. Furthermore, selected MYC-binding sites discovered by the ChIP-Seq analysis described below were validated by real-time DNA-PCR. All primers employed were tested to display an efficiency of approximately 100% (+/−10%). Primer sequences are available from Table S1.

### ChIP-Seq analysis

Approximately 200 ng of ChIP-DNA was used as template for generating an Illumina sequence library (Illumina, San Diego, CA, USA). The DNA was not further size fractionated and directly taken for adaptor ligation, using a standard Illumina genomic library preparation kit. Briefly, DNA was end-repaired using a mix of T4 DNA polymerase, E. coli DNA Pol I large fragment (Klenow polymerase) and T4 polynucleotide kinase. The blunt, phosphorylated ends were treated with Klenow fragment and dATP to yield a protruding 3′-‘A’ base for ligation of Illumina adapters which have a single ‘T’ base overhang at the 3′- end. After adapter ligation fragments of ∼250–350 bp (insert plus adaptor sequences) were isolated from an agarose gel and were PCR amplified with Illumina primers for 15 cycles. The purified DNA was captured on an Illumina flow cell for cluster generation. These libraries were submitted to high-throughput sequencing on the Illumina Genome Analyzer II (GAII).

The resulting sequence reads were mapped to the human reference genome (hg19, GRCh37) using Bowtie [28]. Only reads that mapped uniquely with the Bowtie default setting (http://bowtie-bio.sourceforge.net/manual.shtml#the-n-alignment-mode) for mismatches were considered for further analysis (Bowtie option –m 1).

The detection of genomic regions enriched by ChIP versus input control was conducted with HOMER (v2.6) for each experiment individually [29]. Unique reads were directionally extended in the 3′-direction to a length of 300 base pairs. HOMER fits a local Poisson distribution to the input tags and tests the sequence depth corrected tag counts for being differentially expressed. This effectively removes peaks with low tag counts for which there is a chance that differential enrichment is found simply due to sampling error. Only ChIP regions with a p-value of less than 1e-6 under this local Poisson distribution were considered as putative peaks.

All discovered putative peaks were merged into one list of putative peak regions that were detected in at least one experiment. A matrix containing the number of reads for every experiment in each putative peak region was assembled. DESeq (v1.4.0) was employed to test the number of reads for being differential over all ChIP versus input samples [30]. The 5 different cell lines were considered as biological replicates in order to find common transcription factor binding sites. A negative binomial distribution was fitted to the inputs and tested for being differential in ChIP samples for every peak. The normalization of the number of reads, i.e. the estimation of the size factors for DESeq, was carried out for input controls and ChIP samples separately. Only peak regions with a FDR below 1e-4 were kept for further analysis.

The remaining peaks were ranked by their FDR and annotated with ChIPpeakAnno using Ensembl Biomart employing the Ensembl Genes 59 database and the GCRh37 (hg19) dataset (http://aug2010.archive.ensembl.org/index.html) [31]. The ChIP-Seq files of all experiments are available via the Gene Expression Omnibus (GEO) of the National Center for Biotechnology Information (www.ncbi.nlm.nih.gov/geo/) under the accession number GSE30726.

In order to compare our ChIP-Seq data to previously published MYC-binding sites, we additionally analyzed the data provided by Zeller et al. [24]. For this purpose we converted the genomic hg17 genome coordinates of the published data to hg19 using LiftOver (http://genome.ucsc.edu/cgi-bin/hgLiftOver).

The overlap of genomic intervals determined by ChIP-Seq with genomic features, such as transcriptional start sites or exons, was calculated utilizing RegionMiner (Genomatix Software, Munich, Germany, Version: ElDorado 02-2010, Matrix Library 8.2) [32]. Furthermore, RegionMiner was applied using default settings (i) to calculate the distance correlations with the genomic intervals and the genome-wide experimentally verified transcriptional start regions (TSRs), which were derived from the mapping of selected full length cDNAs (source: GenBank) and CAGE tags (source: FANTOM3, http://fantom3.gsc.riken.jp/) using the oligo capping method, (ii) to determine the occurrence of E-boxes, (iii) to perform an orthologous region search in 6 placental mammalian (Eutheria) species (*Pan troglodytes*, *Macaca mulatta*, *Bos tarus*, *Canis familaris*, *Mus musculus* and *Rattus norvegicus*) and (iv) to determine the overrepresentation (in comparison to the genome and to promoter regions defined as −500 to +100 bp around the start sites of the 159,075 annotated transcripts in ElDorado comprising Refseq, GenBank full-length transcripts and Ensembl records) of the MYC motive as discovered by CoreSearch.

CoreSearch (Genomatix) with default settings was employed for a *de novo* calculation of a MYC-binding motive using the 100 highest ranked genomic intervals [33]. CoreSearch starts with a search for a highly conserved core sequence (called “tuple” in the original publication) which occurs in almost all of the input sequences. In most cases this initial search defines more than one core. Consecutive selection steps are employed in order to reduce the number of core candidates. The final selection is based on maximization of the information content (consensus index), first of the core and then of regions around the core.

### siRNA-mediated MYC knock-down and gene expression analysis

SiRNA-mediated knock-down of MYC was performed employing the BL cell lines Raji, BL41 and Blue1 in order to detect changes of MYC-driven gene expression. For this purpose, the cells were Amaxa-transfected using *MYC* smart pool siRNA and control siRNA (Thermo Scientific/Dharmacon, Erembodegem, Belgium), respectively. Resulting down-regulation of MYC protein expression was monitored by immunoblot analysis [34] using a monoclonal rabbit MYC antibody (clone Y69, Epitomics, Burlingame, USA).

Total RNA of *MYC* siRNA-treated and control siRNA-treated cells was extracted employing the RNeasy Midi kit according to standard protocols (Qiagen, Hilden, Germany). Affymetrix GeneChip analysis (HG-U133A) was performed according to the manufacturer's recommendations, starting with 5 µg total RNA. CEL files were generated with the help of the GCOS 1.3 software (Affymetrix). The CEL files of all experiments are available via the Gene Expression Omnibus (GEO) of the National Center for Biotechnology Information (www.ncbi.nlm.nih.gov/geo/) under the accession number GSE30726.

For analysis of Affymetrix data the expression levels were normalized using VSN [35]. Limma was used to fit a model for the effect of treatment, cell type and experiment batch [36]. Probe sets were tested for differential expression in cell lines with and without *MYC* siRNA treatment using an empirical Bayesian method. P-values were corrected for multiple testing by the method proposed by Benjamini and Hochberg [37]. A corrected p-value of <0.05 and a fold-change >1.2 were used as thresholds.

The differential expression of selected genes was confirmed by Western blotting as previously described [34] with antibodies against CD20 (clone L26, Dako, Glostrup, Denmark), CD79a (clone JCB117, Dako), A20 (own-production), BLNK (sc-8003, Santa Cruz, Santa Cruz, CA USA), CIAP2 (ab32059, Abcam, Cambridge, UK), PAX5 (M7303, Dako), and Actin (ab6276, Abcam) as a control.

### Enrichment analysis of biological annotations

Ensembl annotations (hg19, GRCh37, Ensembl Genes 59) closest to or overlapping with the 7,054 genomic intervals were determined employing the Bioconductor package ChiPpeakAnno (http://www.bioconductor.org/packages/2.5/bioc/html/ChIPpeakAnno.html). The nearest transcription start region (TSR) was used as reference point. By default, the distance is calculated as the distance between the start of the binding site and the TSR that is the gene start for genes located on the forward strand and the gene end for genes located on the reverse strand.

Ensembl annotations next to significant MYC-binding sites (Table S2) and the genes differentially expressed as indicated by our *MYC* siRNA experiments (Table S3) were uploaded to DAVID (The database for annotation visualization and integrated discovery) bioinformatics resources (http://david.abcc.ncifcrf.gov/) [38], [39]. We calculated the most overrepresented (enriched) biological annotations using DAVID default conditions. The total GRCh37 Ensembl gene annotations (Table S2) and the total U133A Affymetrix gene Ids were used as background distribution for the analysis of ChIP-Seq and U133A data, respectively.

## Results

### A genome-wide map of MYC-binding sites in BL

DNA-fragments obtained from five *IG-MYC* translocation positive BL cell lines (Raji, CA46, Blue1, BL41, Ramos) were analyzed after MYC chromatin immunoprecipitation (MYC-ChIP) by next generation sequencing, leading to a total number of more than 16 million and 12 million sequence reads in the MYC immunoprecipitated (ChIP) and input samples, respectively. Alignment to the human reference genome (hg19, GRCh37) using Bowtie default settings mapped 10,882,577 ChIP reads and 8,495,924 input reads uniquely, which were used for further analysis. More details for read numbers and identified peaks at different stages of data processing are given in Table S4.

The software package HOMER (v2.6) [29] was used for peak detection. Experiments were processed individually and all discovered peaks were merged into one list of 19,580 genomic regions that had been detected in at least one experiment. The Bioconductor package DESeq (v1.4.0) [30] was used to test for significant differences in the number of reads between ChIP and input samples across the five cell lines, as described in materials and methods. A list of 7,054 ranked peak regions with an estimated FDR of 1e-4 was kept and mapped to closest or overlapping genes (Ensembl Genes 59 database; GCRh37 dataset), leading to a novel genomic map of BL-specific MYC-binding sites (Table S5). The correlation of the Ensembl annotations with gene biotype (Table S5) revealed that the majority of the 7,054 genomic intervals were associated with protein coding genes (n = 5,793; 82.1%), whereas 565 (8.0%) were mapped to processed transcripts, 202 (2.9%) to pseudogenes and 79 (1.1%) to miRNAs, e.g. the known MYC targets mir-17-92 cluster, hsa-mir-9-3, hsa-let-7a-1 or hsa-mir-29b-2 [40], [41], [42].

**Figure 1:**
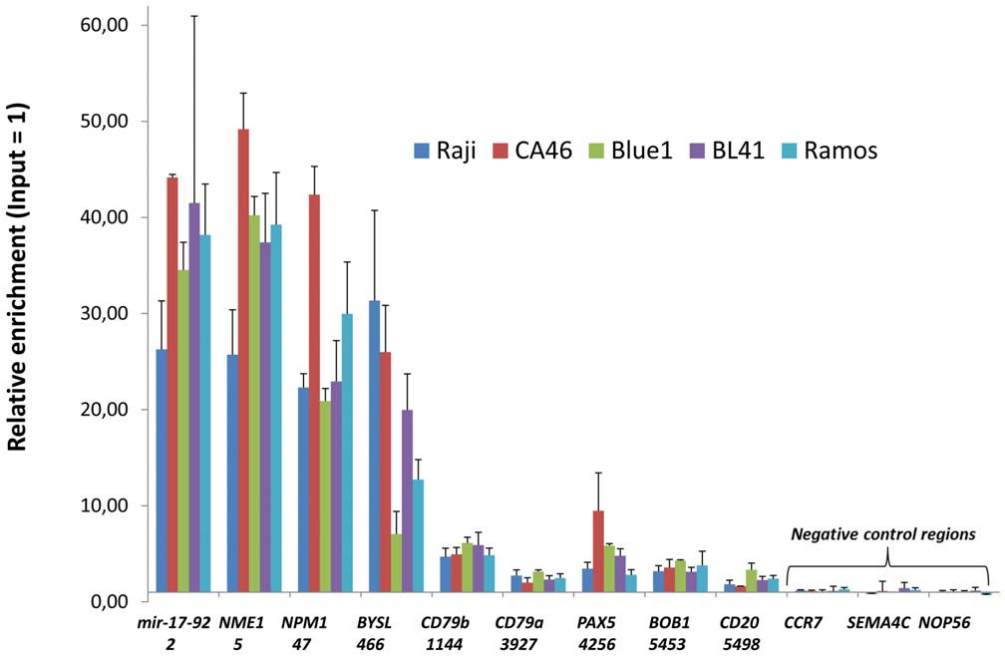
Validation of the ChIP-Seq results by real-time DNA-PCR for selected genomic intervals. Real-time DNA-PCR for 9 selected genes and 3 negative controls was performed with DNA-fragments obtained by ChIP-experiments independent from those used for ChIP-Seq. The ranking position according to Table S5 is indicated under the gene. The enrichment was calculated relative to the ChIP input control.

Real-time DNA-PCR was carried out to verify selected genomic intervals from independently generated ChIP experiments (Figure 1, Figures S1, S2, S3, S4, S5, S6, S7, and S8). Highly ranked genes such as *NME1* (rank position 5) and *NPM1* (rank position 47) revealed a higher enrichment as compared to lower ranked genes such as *BOB1* or *MS4A1* (CD20) (rank position 5453 and 5498, respectively), thereby confirming the reliability of our ChIP-Seq data (Table S5).

**Figure 2:**
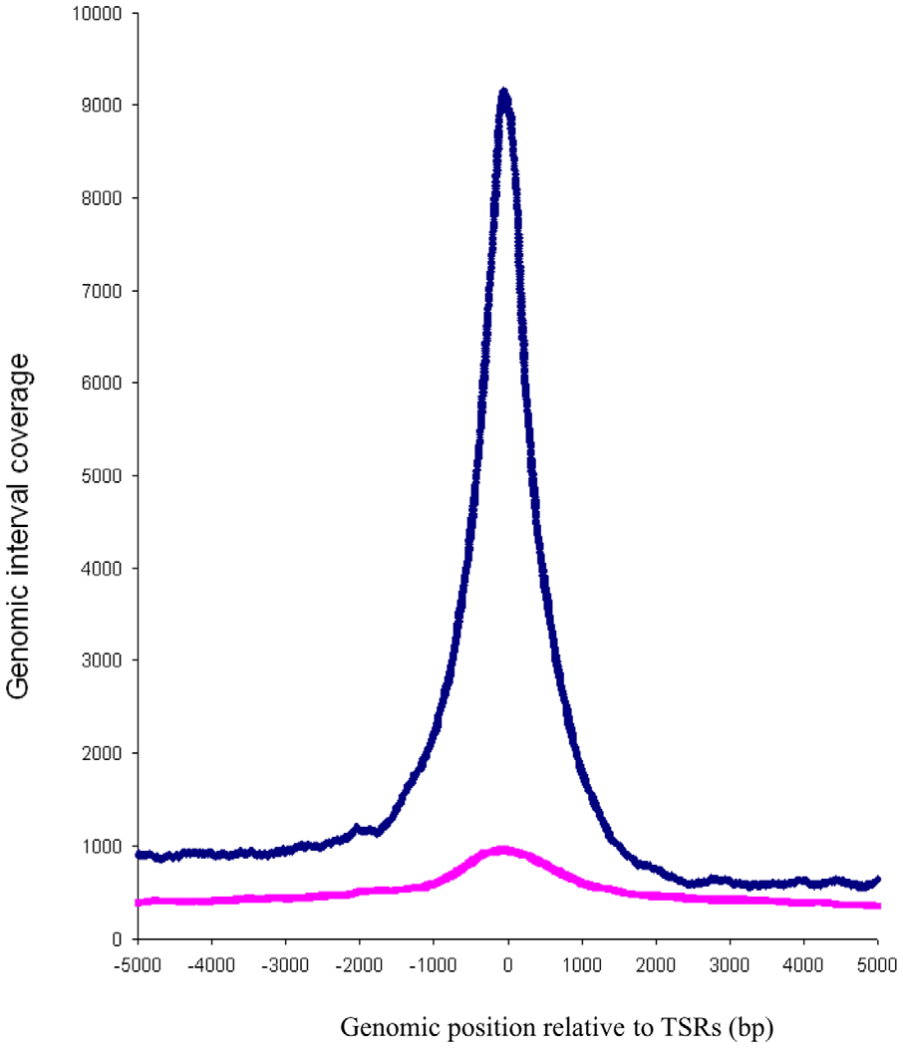
Genome-wide distance correlation between transcriptional start regions (TSRs) and MYC-binding sites. On each TSR, a 10 kbp window was centered and the sum of the genomic intervals representing MYC-binding sites in these regions was determined. Blue line: 7,054 genomic intervals of this ChIP-Seq study. Pink line: 593 ChIP-PET regions from ref [24]. Note: If TSRs are in close proximity to each other, a neighboring genomic interval will have the respective number of correlations with these TSRs. Therefore, more than 7,054 correlations were detectable.

Genome-wide distance correlation revealed that the majority (89.7%) of the identified MYC-binding sites were located in close proximity to transcriptional start sites within +/−1000 bp (Figure 2). Additionally, Genomatix RegionMiner analysis showed that 73.8% of the regions directly overlapped with a transcriptional start region. Only 0.9% of the regions overlapped with repeats (Table 1). The proximity to transcriptional start sites was more pronounced than in a previously published list of 593 MYC binding loci derived from MYC-induced P493-6 cells [24]: 73.8% versus 43.9% transcriptional start regions and 0.9% versus 25.1% repeat regions (Table 1).

**Table 1:** General statistics of the genomic intervals detected by ChIP-Seq analyses in comparison to a ChIP-PET study.

Description	ChIP-Seq analysis	ChIP-PET analysis
Total number of genomic intervals	7,054	590*
Total number of base pairs	2,116,200	858,180
Regions overlap with at least one locus	83.5%	63.9%
Regions overlap with at least one exon	62.9%	34.4%
Regions overlap with at least one intron	65.2%	59.5%
Regions overlap with promoters	67.7%	30.8%
Regions overlap with at least one Transcriptional start region	73.8%	43.9%
Regions overlap with intergenic regions	16.6%	36.6%
Regions overlap with repeats	0.9%	25.1%

The ChIP-Seq data of this study are based on five established BL cell lines, the ChIP-PET data of Zeller and colleagues were produced by analysis of the model cell line P493-6 [24].

*A total of 590 regions were checked ( = 100%), 3 regions were skipped for overlap analysis (>10,000 bp).

Moreover, we found 2,320 exact matches for the canonical E-box sequence CACGTG in our 7,054 genomic intervals (total length: 2,116,200 bp). This corresponds to a 4.6 fold higher enrichment as compared to the 194 occurrences in 590 intervals described by Zeller et al. (814,595 bp; 3 of 593 regions were >10,000 bp and were skipped) [24].

**Figure 3:**
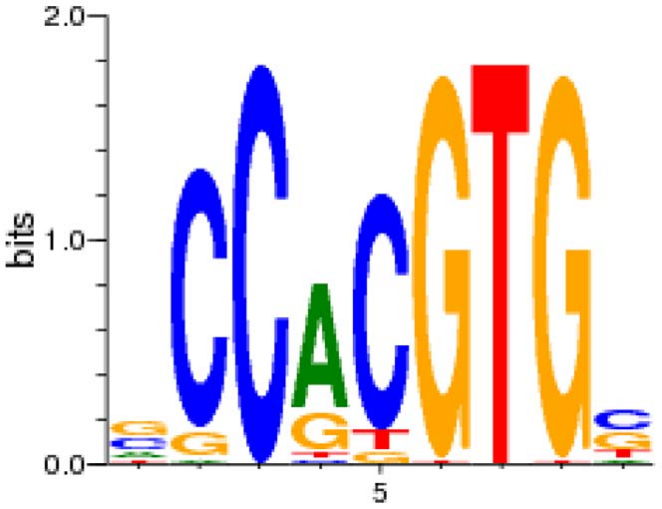
Sequence logo for the de-novo calculated MYC.01-binding site. The transcription factor binding matrix (Table S6) was generated by CoreSearch using the top 100 sequences of MYC genomic intervals (Table S5). A typical E-box motive (CACGTG) was obtained. Highly conserved positions are represented by higher stacks of base symbols A, C, G, and T than less conserved positions. The relative frequencies of the corresponding bases at each position are represented by the relative heights of the symbols within in each stack.

**Figure 4:**
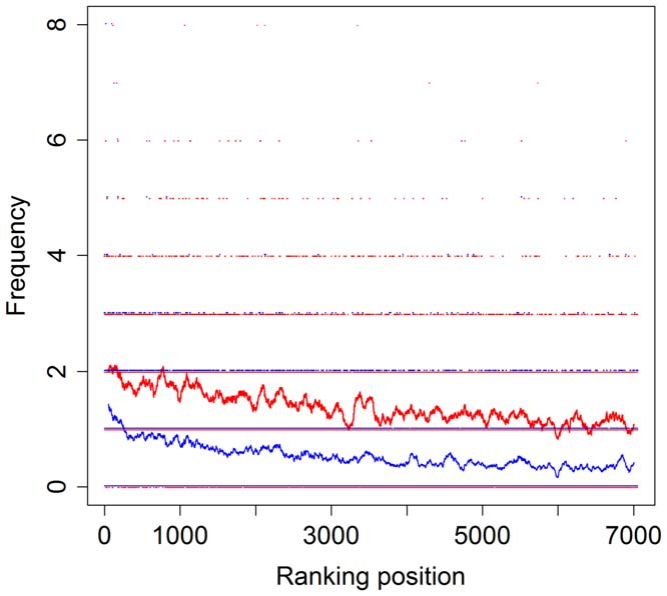
Frequency of MYC-binding sites in comparison to the ranking position of the 7,054 genomic intervals. Y-axis: Frequency of the MYC.01-binding matrix (blue line) and the Genomatix E-box matrix family (red line). X-axis: Ranking position of the 7,054 genomic intervals (Table S5). A moving average with a window size of 100 ranks was used.

The typical MYC-binding E-box motive could also be calculated *de-novo* from our ChIP-Seq data (hereafter called MYC.01-binding site) and is shown in Figure 3. The corresponding matrix is given in Table S6. Genomic intervals with stronger ChIP-Seq signals (i.e. a higher ranking position in Table S5) contained more MYC.01 binding sites compared to genomic intervals with a lower rank position (Figure 4). Performing the same analysis as an independent control with the Genomatix E-box matrix family (generated by Genomatix from independently published experimental evidence) revealed similar results (Figure 4). Genomatix analysis demonstrated that the MYC.01 binding site was enriched 12.71-fold over the genomic background and 5-fold over the promoter background.

5,558 of the 7,054 (78.8%) binding sites are evolutionary conserved with orthologous regions in at least 4 out of 6 placental mammalian genomes (*Pan troglodytes*, *Macaca mulatta*, *Bos tarus*, *Canis familaris*, *Mus musculus* and *Rattus norvegicus*; Table S5).

### Enrichment of functional classes of genes

Gene set enrichment analysis using the DAVID software [38], [39] showed that a set of genes involved in RNA metabolism was most strongly enriched in our list of MYC targets genes in BL (Table S7). In line with this finding, genes encoding proteins with specific RNA or DNA-binding domains (e.g. RNA recognition motives (RRM) and helicase domains (HELICc)) were significantly overrepresented. Moreover, gene sets associated with ribosomal biogenesis, spliceosome, aminoacyl-tRNA biosynthesis or pyrimidine and purine metabolism or involved in the cell cycle were frequent targets of MYC binding. Interestingly, genes associated with histone acetyltransferase and methyltransferase complexes were also accumulated suggesting a function of MYC in the control of epigenetic mechanisms. This notion is reinforced by the enrichment of genes containing plant homeodomain (PHD) finger and the SET domains which are supposed to be involved in chromatin-mediated transcriptional regulation [43]. Strikingly, the genes of the Polycomb group (PcG) proteins EZH1 and EZH2, which are histone methyltransferases catalyzing the repressive trimethylation of lysine 27 on histone H3 (H3K27me3), were among the MYC target genes [44]. In addition 12 further PcG complex protein members (*EED*, *BMI1*, *PHF1*, *MTF2*, *PHF19*, *L3MBTL*, *PHC3*, *SCMH1*, *PCGF1*, *CBX4/6/7*) displayed MYC binding in BL. 10 of these (71.4%) had orthologous regions in at least 4 of 6 placental mammals (Table S5) [44].

Especially remarkable was our finding that genes involved in B-cell receptor signaling as well as B-cell differentiation and activation (e.g. *CD19*, *CD20* (*MS4A1*), *CD21* (*CR2*), *CD22*, *CD79a/b*, *SYK*, *LYN*, *BLK*, *BLNK*, *BOB1* (*POU2AF1*) and *PAX5*) were identified as active MYC-binding sites (Table S5). Since MYC is regarded as an important oncogene in several types of malignant lymphomas (including BL), this new observation might be of high relevance for the understanding of the biology of these lymphomas. This is further underscored by the result that Ensembl annotations related to evolutionary conserved intervals in at least 4 of 6 placental mammals revealed also an enrichment of the B-cell receptor pathway (Table S8).

### siRNA-mediated silencing of MYC in BL cell lines

**Figure 5:**
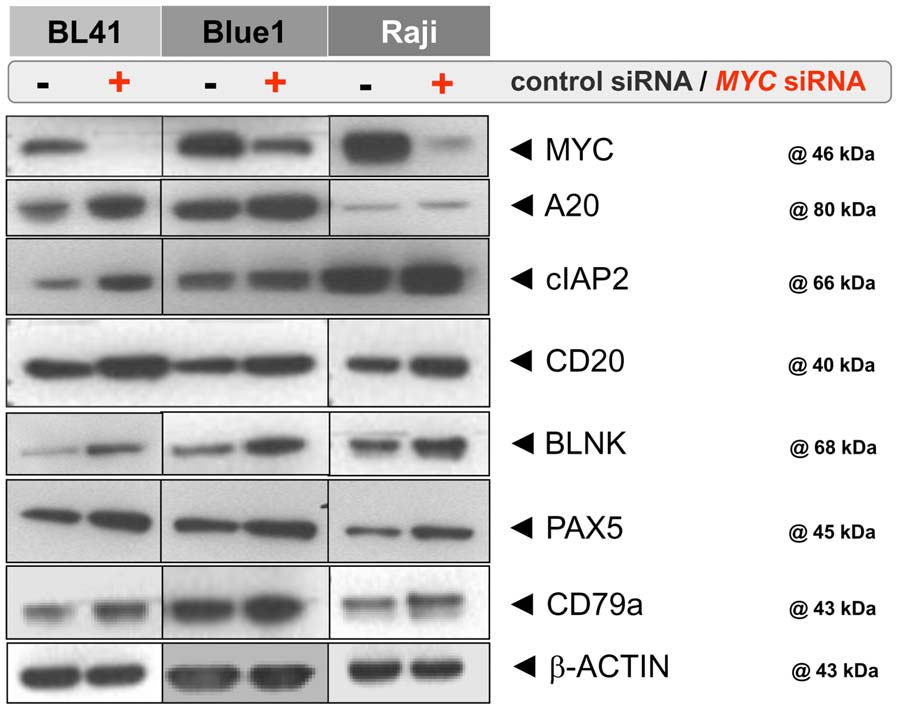
Western Blot analysis after siRNA-mediated MYC knock-down in 3 BL cell lines. An up-regulation of selected NF-kB pathway proteins (A20, CIAP2) and B-cell genes (CD20, BLNK, PAX5, CD79a) was detected by Western blot analysis of protein extracts derived from control siRNA-treated and MYC siRNA-treated BL cell lines. β-Actin (one example shown) was used as loading control.

We employed *MYC* siRNA to analyze the impact of MYC on gene expression. Treatment of three BL cell lines (Raji, BL41, Blue1) with *MYC*-specific siRNAs resulted in a reduction of the MYC protein of approx. 75% (Figure 5). Gene expression profiling using Affymetrix GeneChips (U133A) of the siRNA-treated and control siRNA-treated cell lines revealed that upon MYC silencing 168 and 17 genes were up-regulated, whereas 828 and 87 genes were significantly down-regulated with fold changes of 1.2 and 1.4, respectively, and with a corrected p-value<0.05 (Table S3).

In line with our ChIP-Seq data, DAVID gene set enrichment analysis revealed that down-regulated genes were related to cell cycle, as well as to transcriptional and translational processes (Figure S9, Table S7 and S9). Most interestingly, several of the up-regulated genes were related to the B-cell receptor signaling pathway, B-cell activation and antigen processing and presentation (Table S9).

Western blot analysis supported the RNA data: a moderately increased protein expression was observed for several B-cell antigens (CD20, CD79a, BLNK, PAX5), as well as for NF-kB associated proteins (A20, CIAP2), which are down-stream components of the B-cell receptor pathway (Figure 5).

## Discussion

The understanding of MYC biology is of paramount importance to elucidate its role in the pathogenesis of Burkitt lymphoma (BL), a disease characterized by a consistent high MYC protein expression due to a genomic translocation. Therefore we undertook a comprehensive approach to identify MYC-binding sites by chromatin immunoprecipitation with subsequent deep sequencing (ChIP-Seq). This led to a ranked list of 7,054 MYC-binding genomic intervals in BL (Table S5).

ChIP-Seq is a complex technique which requires further independent validation to confirm the reliability of the data. Our ChIP-Seq data are supported by three independent lines of evidence: (i) Confirmation of the exact MYC-binding position in the promoters of previously published MYC target genes (e.g. *NPM1*, *NME1*) and identification of MYC-binding sites associated with miRNAs known to be regulated by MYC (e.g. the mir-17-92 cluster, hsa-mir-9-3, hsa-let-7a-1 or hsa-mir-29b-2) (Figure 1, Table S5) [24], [40], [41], [42]. (ii) The vast majority of the MYC-binding sites occurred at transcriptional start sites in line with the role of MYC as a transcription factor (Figure 2). (iii) The typical MYC-binding E-box motive could be calculated de-novo from our ChIP-Seq data (Figure 3).

Our genome-wide approach largely extends previous MYC ChIP-analyses from one BL cell line using promoter tiling arrays (ChIP-on-chip) covering only 4,839 proximal promoters [23]. In a further pioneer study MYC-binding sites were assessed by ChIP followed by pair-end ditag sequencing (ChIP-PET) in one artificial human BL model cell line (P493-6) after synthetic induction of MYC [24]. This approach revealed 1,143,746 PET units, 691,966 of which had a single mapping location in the human hg17 genome finally resulting in only 593 MYC-binding sites (PET clusters with at least 3 overlapping PETs) [24]. In contrast, our 7,054 MYC-binding genomic intervals derived from 5 BL cell lines allow a much more precise and representative consideration of the MYC landscape in BL due to a much higher coverage of MYC-binding sites.

Gene set enrichment analysis using the Ensembl annotations related to MYC binding (Table S5) revealed enrichments for ribosomal biogenesis, and pyrimidine and purine metabolism (Table S7). This confirms previous findings and further underlines the plausibility of our data [45], [46]. Ribosomal biogenesis is a primary function of the nucleolus and globally essential for RNA translation [46]. This process is controlled by the ribosomal biogenesis (RiBi) genes [46]. Recently some RiBi genes were identified as MYC target genes [46]. We add further evidence to the important role of MYC in ribosomal biogenesis by demonstrating the binding of MYC to 68 of 87 (78.2%) genes in the KEGG ribosome pathway in BL (Table S7). Interestingly, a specific RiBi core-promoter signature which is partially composed of E-boxes was found to be conserved from fly to man [47]. However, the nematode genome is a remarkable exception from this rule since it appears to have secondarily lost its *myc* gene along with the E-box containing RiBi core-promoter signature [47]. In contrast to other animals, nematodes have a very specific total cell number, and it is appealing to speculate that the lack of *MYC* is a prerequisite for maintenance of this precise cell number, most likely by a lack of MYC-driven cell proliferation.

Another important finding of our study is the binding of MYC to many Polycomb group (PcG) genes in BL. PcG proteins were identified in Drosophila more than 30 years ago as regulators of anterior-posterior body patterning through the repression of *Hox* genes [44]. One of the first indications that PcG proteins play a role in cancer was the identification of *BMI1* as MYC-collaborating oncogene [48], [49]. Here we establish another strong linkage between PcG and MYC by showing that 14 PcG complex genes are MYC targets including *EZH1/2*, *EED* and *BMI1*. Given the important role of PcG in B-cell development, lymphomagenesis, and tumor stem cell development [44] it will be interesting to further analyze the relation between MYC, PcG and PcG target genes.

An unexpected observation based on our ChIP-Seq experiments, was the finding of many B-cell genes (*CD19*, *CD20* (*MS4A1*), *CD21* (*CR2*), *CD22*, *PAX5*, *SYK*, *LYN*, *BLK*, *BLNK*, *BOB1* (*POU2AF1*), *CD79a/b*) as active MYC target genes (Table S5, Figure 1). Previously, merely single B-cell genes such as *CD79b* were suggested as MYC target genes [23], [24], [50]. Our data considerably extend the list of B-cell genes targeted by MYC including one of the prominent B-cell transcription factors, namely PAX5. *PAX5*, which harbors 4 MYC binding motifs, is one of the master regulators for B-cell development and for the maintenance of the B-cell phenotype of mature B-cells (Table S2). This raises the question of whether MYC is an important transcription factor involved in the B-cell differentiation, a notion which is very much supported by recent findings derived from early murine B-cell development [51]. Moreover, gene set enrichment analysis based on our 5,558 MYC genomic intervals with orthologous regions in at least 4 of 6 placental mammals showed significantly enriched B-cell receptor pathway targets and B-cell development related GO terms (Table S8).

The role of MYC in the B-cell development and B-cell function is further underlined by our MYC knock-down experiments where we observed an up-regulation of many genes well known for their function in B-cells (Figure 5, Table S3 and S9). The up-regulation of B-cell relevant genes as the consequence of MYC silencing is additionally supported by recent results derived from the BL model cell line P493-6 [52] since B-cell related genes such as *CD79a/b*, *CD19*, *CD20*, *CD22*, *CD72*, as well as most HLA molecules were down-regulated after MYC induction [52]. This down-regulation of HLA upon MYC induction suggests an important role of MYC as an immunomodulatory factor (Table S3 and S9). HLA molecules play an essential role in antigen presentation of the BL cells, and down-regulation of HLA molecules by MYC can lead to a lack of immunogenicity in BL cells [53].

Taken together, the 7,054 MYC-binding sites identified by our ChIP-Seq approach greatly extend the current knowledge regarding MYC binding in BL and further elucidate the complexity of the very comprehensive role of MYC in many regulatory networks. Especially our discovery of MYC as a transcription factor involved in B-cell differentiation and B-cell signaling is of great importance for a better understanding of MYC-driven lymphomas such as BL.

## Supporting Information

Figure S1 **MYC-binding sites in the** ***NME1*** **gene.** ChIP-Seq reads obtained after MYC ChIP-Seq and from input controls analyzing 5 BL cell lines (BL41, Blue1, CA46, Ramos, Raji) are illustrated for the 5′- ends of the *NME1* gene by using the UCSC genome browser (http://genome.ucsc.edu/). Reads in red map to the forward strand and blue reads to the reverse strand. The location of real-time DNA-PCR (Table S1) is schematically indicated above the gene annotations as well as the genomic intervals identified by bioinformatic analysis (Table S5).
(PDF)

Figure S2 **MYC-binding sites in the** ***NPM1*** **gene.** ChIP-Seq reads obtained after MYC ChIP-Seq and from input controls analyzing 5 BL cell lines (BL41, Blue1, CA46, Ramos, Raji) are illustrated for the 5′- ends of the *NPM1* gene by using the UCSC genome browser (http://genome.ucsc.edu/). Reads in red map to the forward strand and blue reads to the reverse strand. The location of real-time DNA-PCR (Table S1) is schematically indicated above the gene annotations as well as the genomic intervals identified by bioinformatic analysis (Table S5).
(PDF)

Figure S3 **MYC-binding sites in the** ***BYSL*** **gene.** ChIP-Seq reads obtained after MYC ChIP-Seq and from input controls analyzing 5 BL cell lines (BL41, Blue1, CA46, Ramos, Raji) are illustrated for the 5′- ends of the *BYSL* gene by using the UCSC genome browser (http://genome.ucsc.edu/). Reads in red map to the forward strand and blue reads to the reverse strand. The location of real-time DNA-PCR (Table S1) is schematically indicated above the gene annotations as well as the genomic intervals identified by bioinformatic analysis (Table S5).
(PDF)

Figure S4 **MYC-binding sites in the** ***PAX5*** **gene.** ChIP-Seq reads obtained after MYC ChIP-Seq and from input controls analyzing 5 BL cell lines (BL41, Blue1, CA46, Ramos, Raji) are illustrated for the 5′- ends of the *PAX5* gene by using the UCSC genome browser (http://genome.ucsc.edu/). Reads in red map to the forward strand and blue reads to the reverse strand. The location of real-time DNA-PCR (Table S1) is schematically indicated above the gene annotations as well as the genomic intervals identified by bioinformatic analysis (Table S5).
(PDF)

Figure S5 **MYC-binding sites in the** ***MS4A1*** **gene.** ChIP-Seq reads obtained after MYC ChIP-Seq and from input controls analyzing 5 BL cell lines (BL41, Blue1, CA46, Ramos, Raji) are illustrated for the 5′- ends of the *MS4A1* gene by using the UCSC genome browser (http://genome.ucsc.edu/). Reads in red map to the forward strand and blue reads to the reverse strand. The location of real-time DNA-PCR (Table S1) is schematically indicated above the gene annotations as well as the genomic intervals identified by bioinformatic analysis (Table S5).
(PDF)

Figure S6 ***NOP56*** **intron 8 (negative control).** ChIP-Seq reads obtained after MYC ChIP-Seq and from input controls analyzing 5 BL cell lines (BL41, Blue1, CA46, Ramos, Raji) are illustrated by using the UCSC genome browser (http://genome.ucsc.edu/). Reads in red map to the forward strand and blue reads to the reverse strand. The location of real-time DNA-PCR (Table S1) is schematically indicated above the gene annotations.
(PDF)

Figure S7 **CCR7 (negative control).** ChIP-Seq reads obtained after MYC ChIP-Seq and from input controls analyzing 5 BL cell lines (BL41, Blue1, CA46, Ramos, Raji) are illustrated by using the UCSC genome browser (http://genome.ucsc.edu/). Reads in red map to the forward strand and blue reads to the reverse strand. The location of real-time DNA-PCR (Table S1) is schematically indicated above the gene annotations.
(PDF)

Figure S8 **SEMA4C (negative control).** ChIP-Seq reads obtained after MYC ChIP-Seq and from input controls analyzing 5 BL cell lines (BL41, Blue1, CA46, Ramos, Raji) are illustrated by using the UCSC genome browser (http://genome.ucsc.edu/). Reads in red map to the forward strand and blue reads to the reverse strand. The location of real-time DNA-PCR (Table S1) is schematically indicated above the gene annotations.
(PDF)

Figure S9 **Significantly enriched KEGG pathways detected by ChIP-Seq analysis (Table S7) in relation to KEGG pathways detected by siRNA-mediated knock-downs of MYC in BL cell lines followed by gene expression profiling.**
(TIF)

Table S1 Primer sequences for real-time DNA-PCRs.
(XLS)

Table S2 Complete list of Ensembl annotations (hg19, GRCh37, Ensembl Genes 59) annotated with the number of detected MYC-binding sites for each Ensembl annotation.
(XLS)

Table S3 Differential gene expression after siRNA mediated knock-down of MYC. Three BL cell lines (Raji, BL41 and Blue1) were analyzed employing U133A Affymetrix GeneChip hybridization.
(XLS)

Table S4 Number of reads in the Fastq files for all MYC ChIP-Seq and input samples mapped to the human hg19 reference genome. The number of reads within the putative peaks discovered by HOMER and within the final list of 7,054 merged peaks (DESeq) is shown in the far right column.
(DOC)

Table S5 7,054 peak regions (i.e. MYC-binding sites) with a FDR below 1e-4 are ranked by their FDR. Their closest or overlapping gene annotation (hg19, GRCh37, Ensembl Genes 59) is given.
(XLS)

Table S6 MYC.01 Transcription factor binding matrix. The MYC.01 binding matrix was generated de novo by CoreSearch using the top 100 sequences in Table S5. The matrix results from the alignment of highly similar short sequence motifs in 98 of these input sequences; frequencies of A, C, G, and T at each alignment position are given. The Consensus Index (Ci) is a measure for the conservation of each position and is used for weighting each position's contribution to the overall score when searching for matrix matches in a target sequence. A typical E-box motive (CACGTG) was obtained (Figure 3).
(XLS)

Table S7 Biological annotations associated with genes next to the 7,054 genomic intervals (i.e. MYC-binding sites). Interesting terms are highlighted in red.
(XLS)

Table S8 Biological annotations associated with 5,558 of the 7,054 genomic intervals with orthologous regions in at least 4 of 6 mammalian species. Genomatix RegionMiner with default parameters was employed for the search of orthologous genomic regions. Interesting terms are highlighted in red.
(XLS)

Table S9 Biological annotations associated with genes differentially expressed after siRNA-mediated MYC knock-down in 3 BL cell lines (Raji, BL41, Blue1). Interesting terms are highlighted in red.
(XLS)
