# Supplementary figures and images for: Deep Sequencing of MYC DNA-Binding Sites in Burkitt Lymphoma
Source: PLoS One. 2011 Nov 10;6(11):e26837. doi: 10.1371/journal.pone.0026837 (PMC3213110; doi:10.1371/journal.pone.0026837)

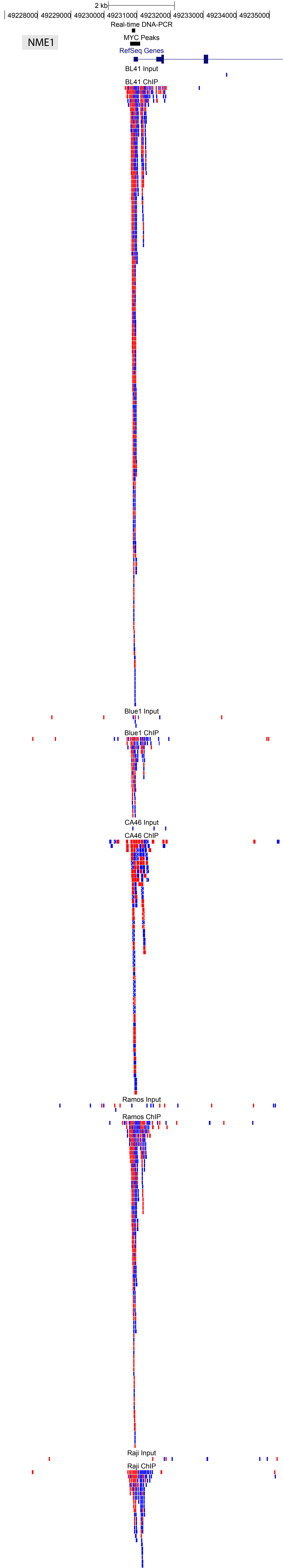

Supplement: Figure S1 — MYC-binding sites in the NME1 gene. ChIP-Seq reads obtained after MYC ChIP-Seq and from input controls analyzing 5 BL cell lines (BL41, Blue1, CA46, Ramos, Raji) are illustrated for the 5′- ends of the NME1 gene by using the UCSC genome browser (http://genome.ucsc.edu/). Reads in red map to the forward strand and blue reads to the reverse strand. The location of real-time DNA-PCR (Table S1) is schematically indicated above the gene annotations as well as the genomic intervals identified by bioinformatic analysis (Table S5). (PDF) [file pone.0026837.s001.pdf]

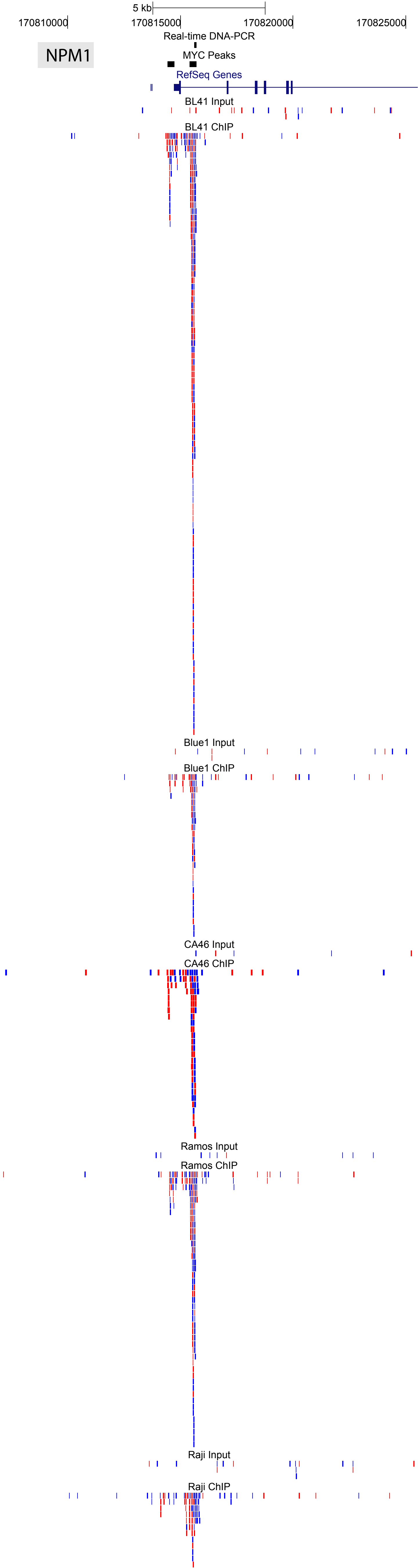

Supplement: Figure S2 — MYC-binding sites in the NPM1 gene. ChIP-Seq reads obtained after MYC ChIP-Seq and from input controls analyzing 5 BL cell lines (BL41, Blue1, CA46, Ramos, Raji) are illustrated for the 5′- ends of the NPM1 gene by using the UCSC genome browser (http://genome.ucsc.edu/). Reads in red map to the forward strand and blue reads to the reverse strand. The location of real-time DNA-PCR (Table S1) is schematically indicated above the gene annotations as well as the genomic intervals identified by bioinformatic analysis (Table S5). (PDF) [file pone.0026837.s002.pdf]

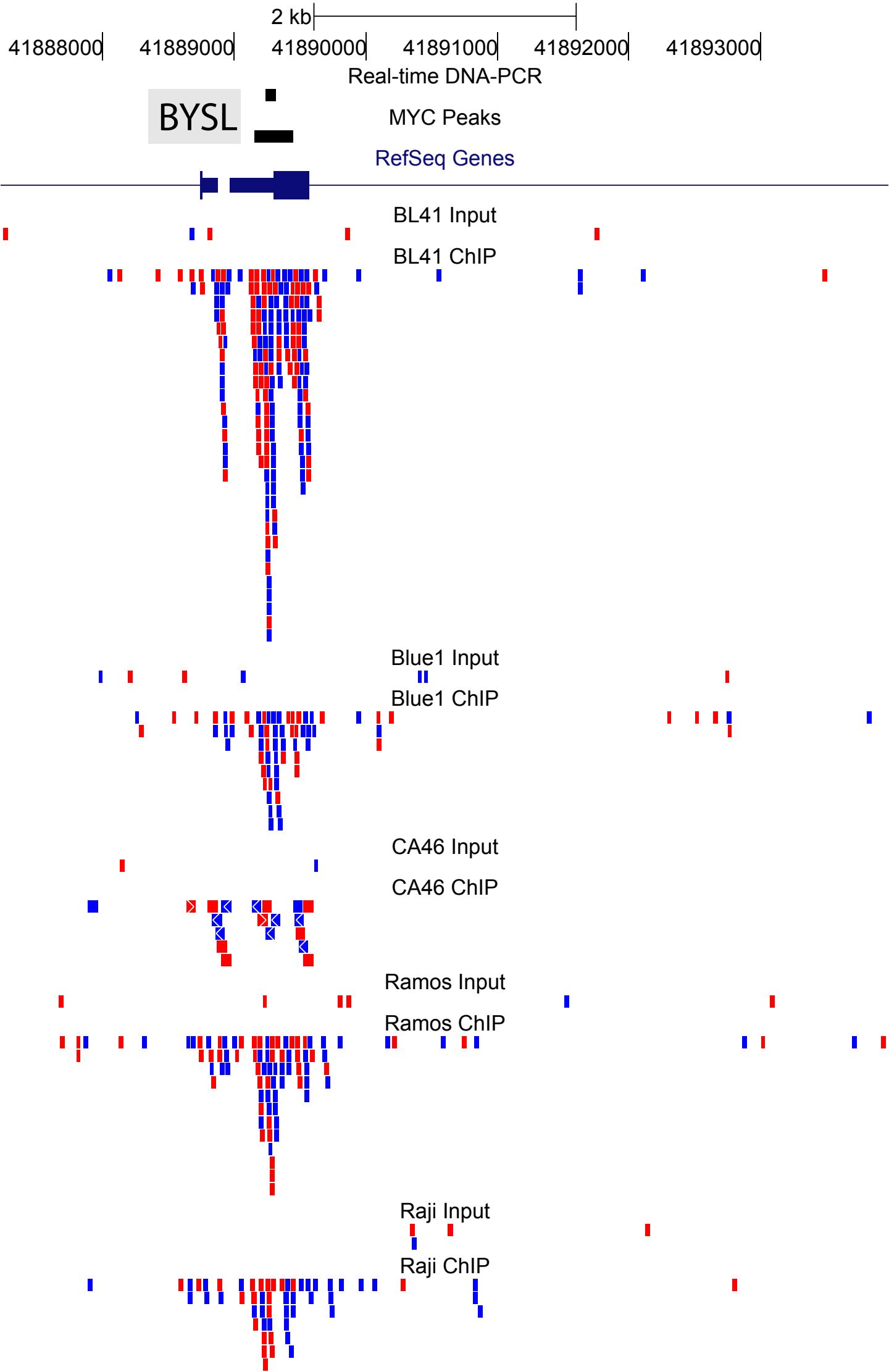

Supplement: Figure S3 — MYC-binding sites in the BYSL gene. ChIP-Seq reads obtained after MYC ChIP-Seq and from input controls analyzing 5 BL cell lines (BL41, Blue1, CA46, Ramos, Raji) are illustrated for the 5′- ends of the BYSL gene by using the UCSC genome browser (http://genome.ucsc.edu/). Reads in red map to the forward strand and blue reads to the reverse strand. The location of real-time DNA-PCR (Table S1) is schematically indicated above the gene annotations as well as the genomic intervals identified by bioinformatic analysis (Table S5). (PDF) [file pone.0026837.s003.pdf]

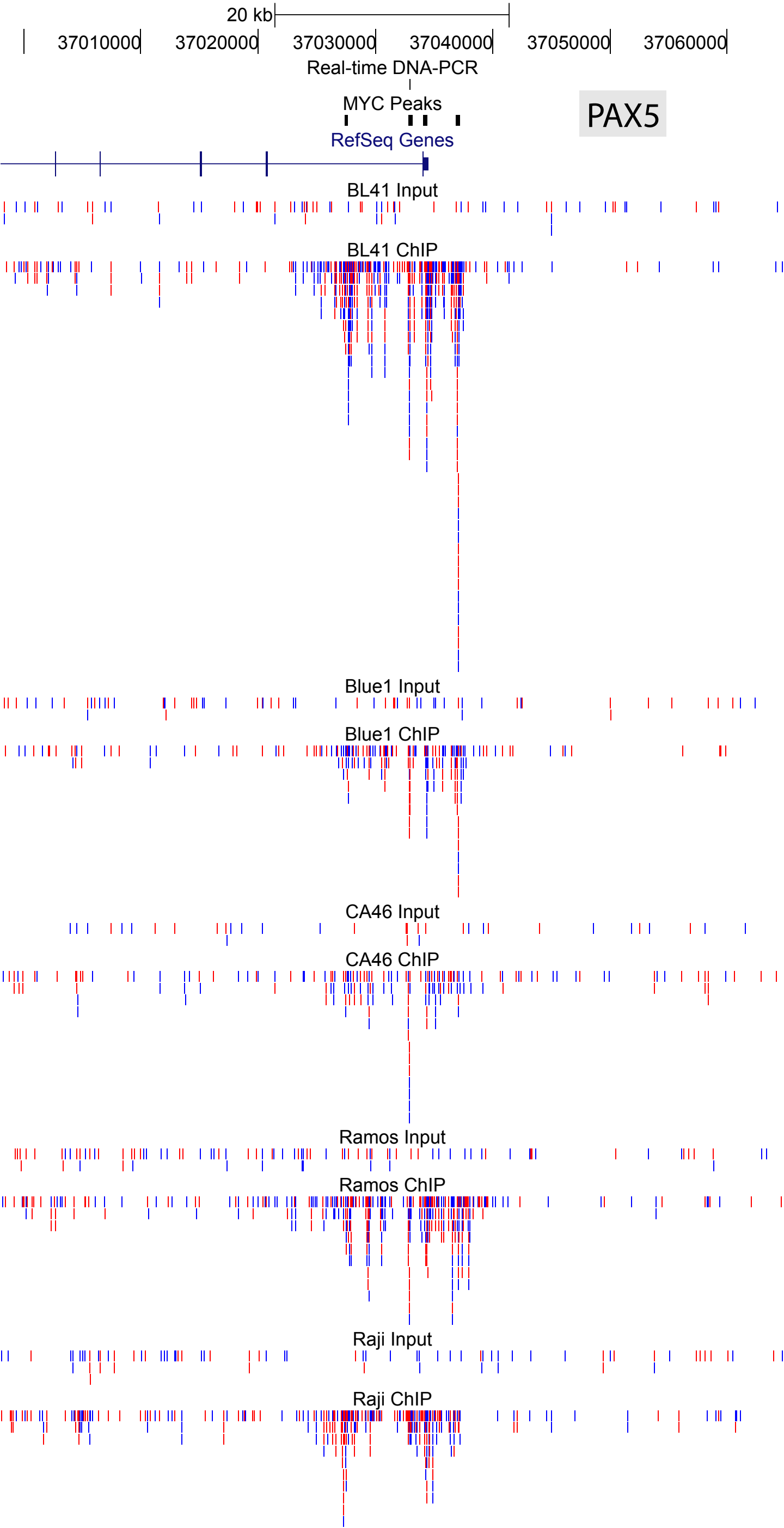

Supplement: Figure S4 — MYC-binding sites in the PAX5 gene. ChIP-Seq reads obtained after MYC ChIP-Seq and from input controls analyzing 5 BL cell lines (BL41, Blue1, CA46, Ramos, Raji) are illustrated for the 5′- ends of the PAX5 gene by using the UCSC genome browser (http://genome.ucsc.edu/). Reads in red map to the forward strand and blue reads to the reverse strand. The location of real-time DNA-PCR (Table S1) is schematically indicated above the gene annotations as well as the genomic intervals identified by bioinformatic analysis (Table S5). (PDF) [file pone.0026837.s004.pdf]

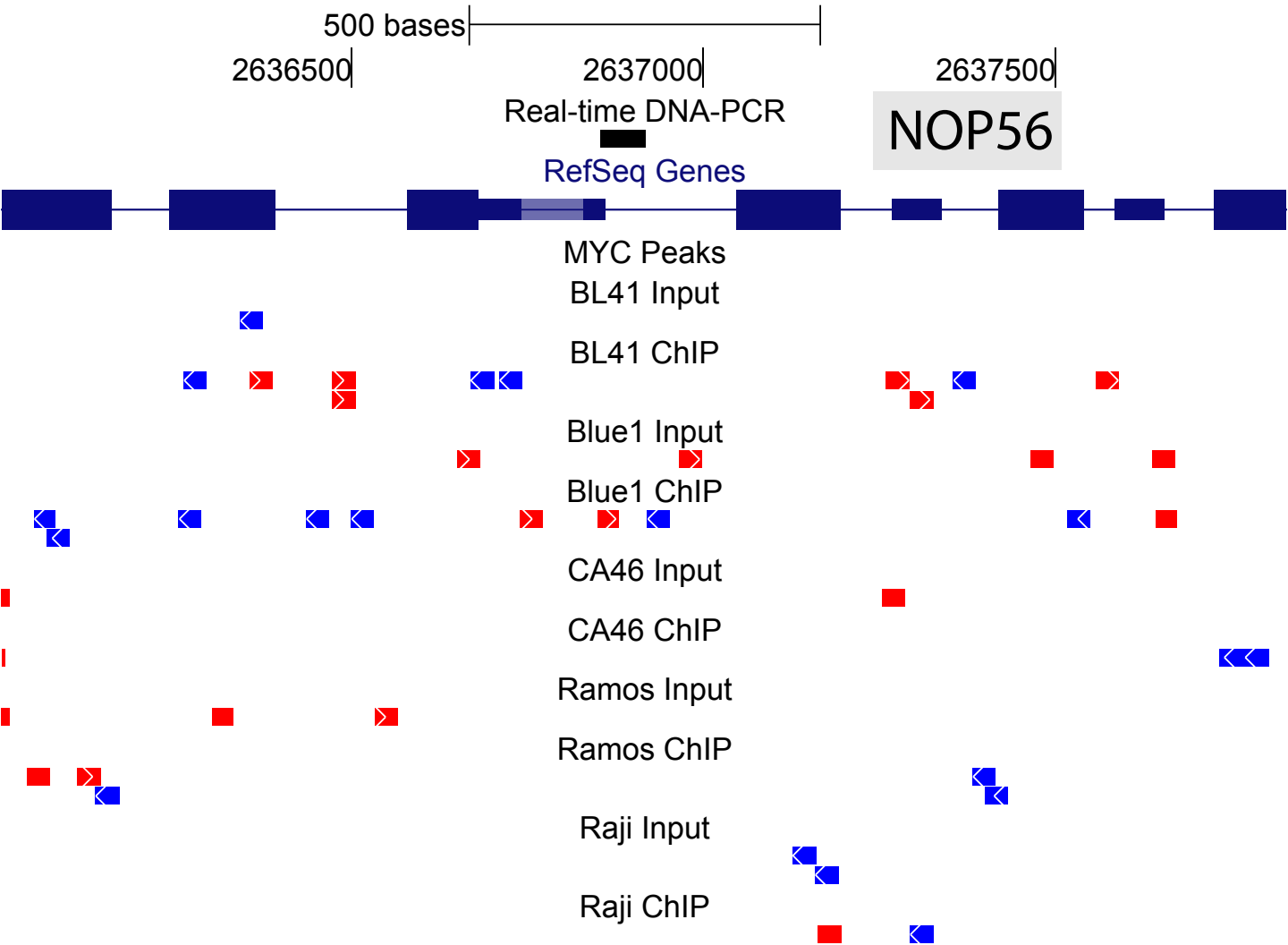

Supplement: Figure S6 — NOP56 intron 8 (negative control). ChIP-Seq reads obtained after MYC ChIP-Seq and from input controls analyzing 5 BL cell lines (BL41, Blue1, CA46, Ramos, Raji) are illustrated by using the UCSC genome browser (http://genome.ucsc.edu/). Reads in red map to the forward strand and blue reads to the reverse strand. The location of real-time DNA-PCR (Table S1) is schematically indicated above the gene annotations. (PDF) [file pone.0026837.s006.pdf]

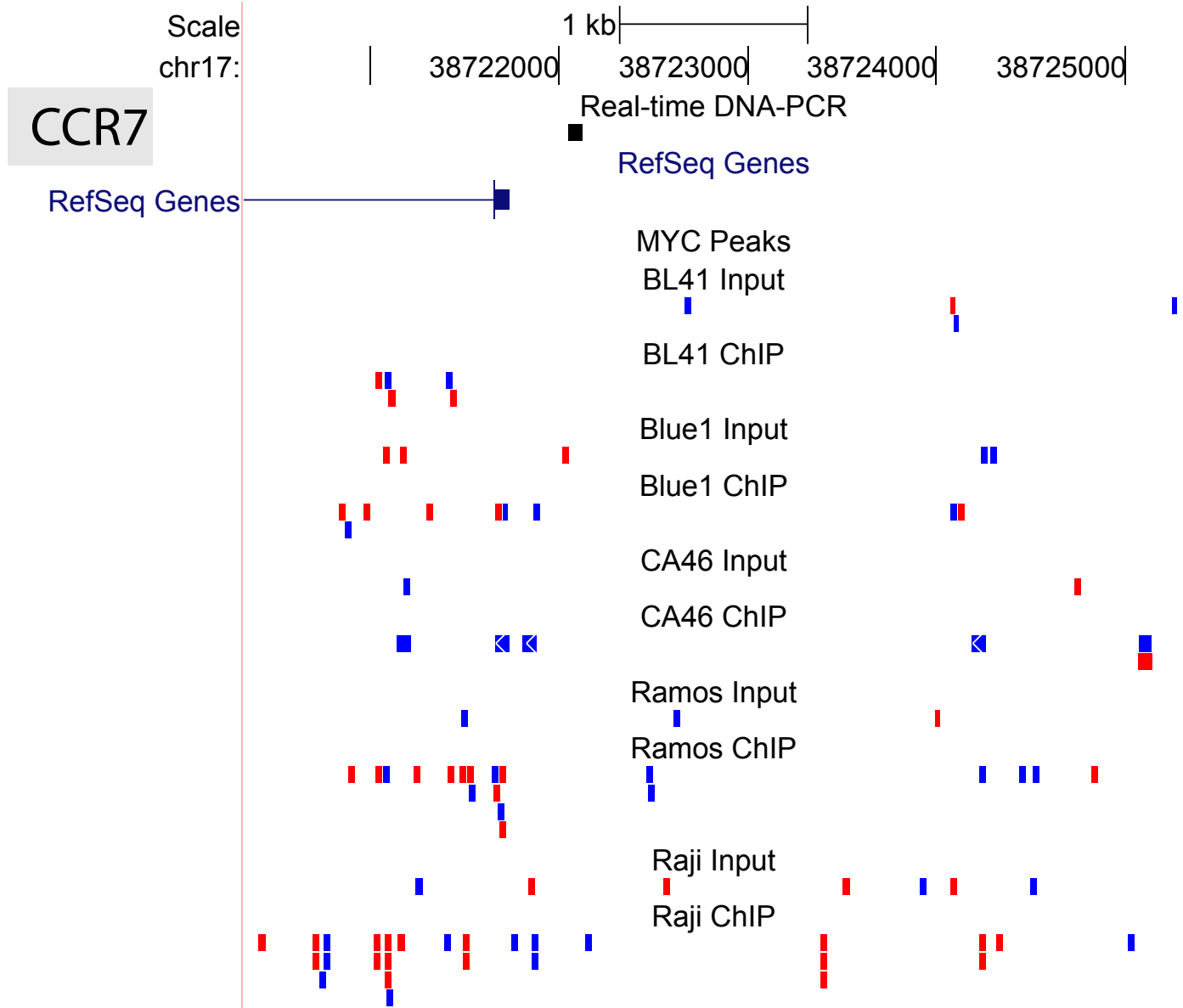

Supplement: Figure S7 — CCR7 (negative control). ChIP-Seq reads obtained after MYC ChIP-Seq and from input controls analyzing 5 BL cell lines (BL41, Blue1, CA46, Ramos, Raji) are illustrated by using the UCSC genome browser (http://genome.ucsc.edu/). Reads in red map to the forward strand and blue reads to the reverse strand. The location of real-time DNA-PCR (Table S1) is schematically indicated above the gene annotations. (PDF) [file pone.0026837.s007.pdf]

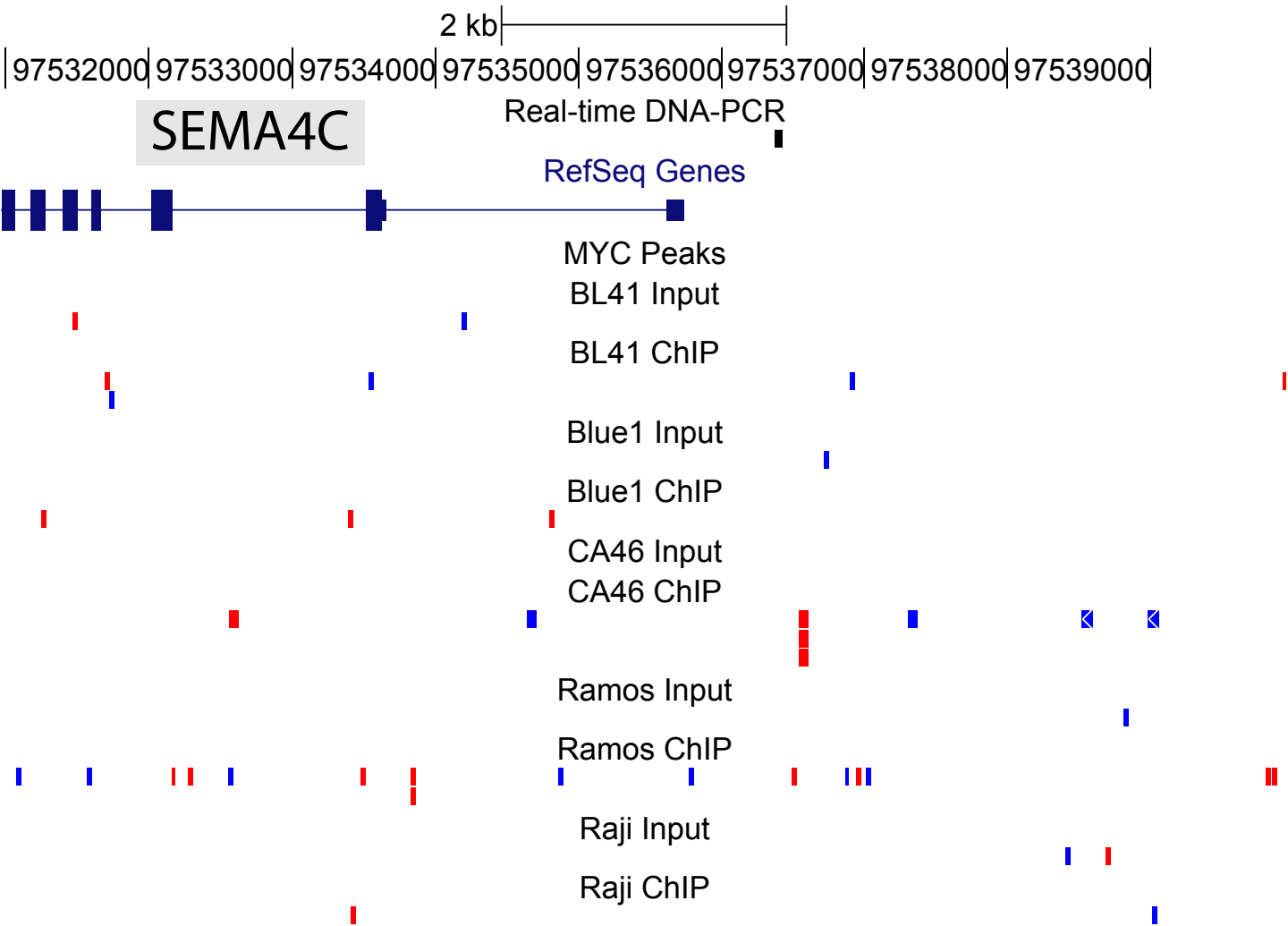

Supplement: Figure S8 — SEMA4C (negative control). ChIP-Seq reads obtained after MYC ChIP-Seq and from input controls analyzing 5 BL cell lines (BL41, Blue1, CA46, Ramos, Raji) are illustrated by using the UCSC genome browser (http://genome.ucsc.edu/). Reads in red map to the forward strand and blue reads to the reverse strand. The location of real-time DNA-PCR (Table S1) is schematically indicated above the gene annotations. (PDF) [file pone.0026837.s008.pdf]

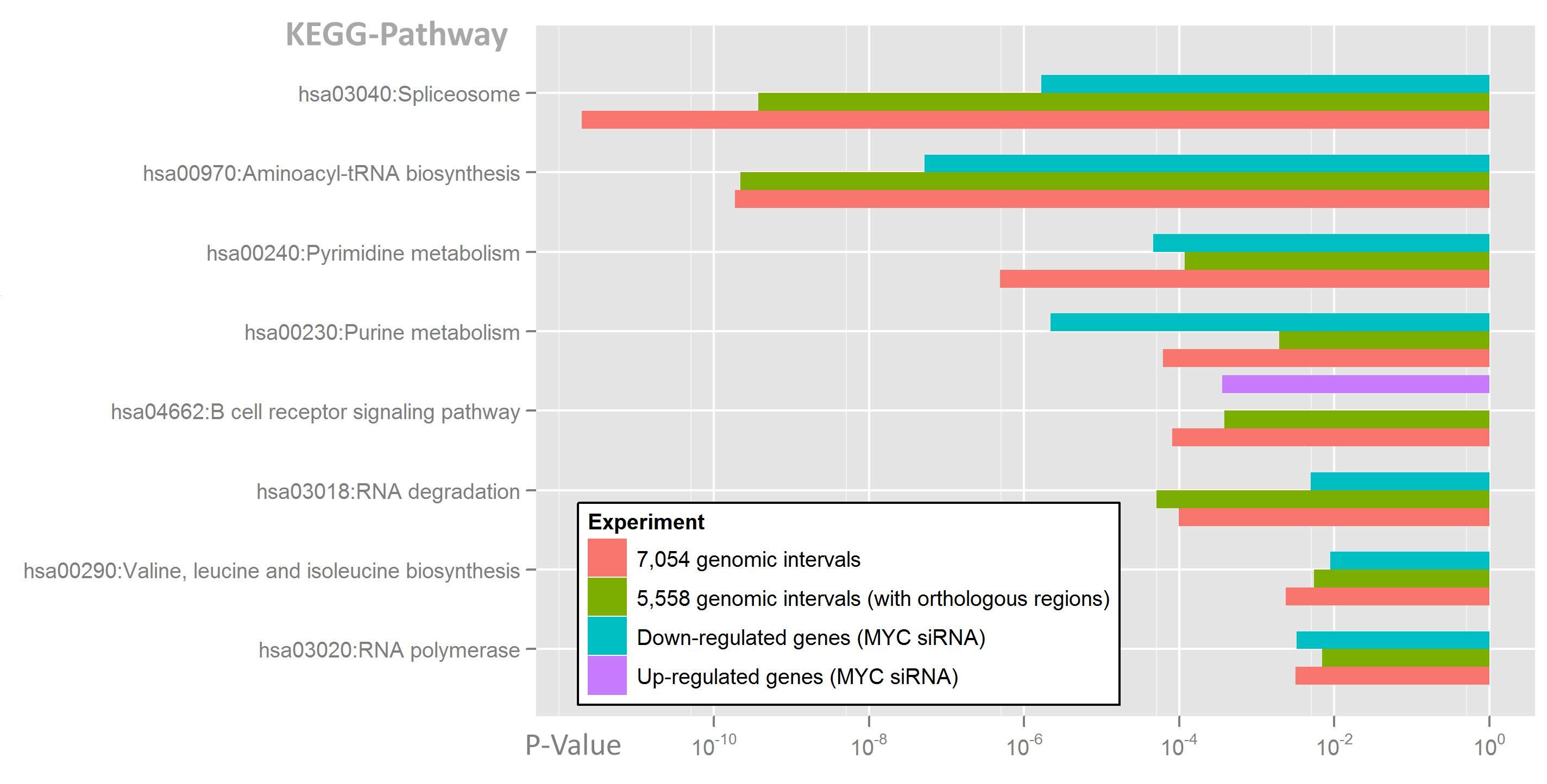

Supplement: Figure S9 — Significantly enriched KEGG pathways detected by ChIP-Seq analysis (Table S7) in relation to KEGG pathways detected by siRNA-mediated knock-downs of MYC in BL cell lines followed by gene expression profiling. (TIF) [file pone.0026837.s009.tif]
