# Supplementary material for: Deep Sequencing of MYC DNA-Binding Sites in Burkitt Lymphoma
Source: PLoS One. 2011 Nov 10;6(11):e26837. doi: 10.1371/journal.pone.0026837 (PMC3213110; doi:10.1371/journal.pone.0026837)
Supplement: Figure S5 — MYC-binding sites in the MS4A1 gene. ChIP-Seq reads obtained after MYC ChIP-Seq and from input controls analyzing 5 BL cell lines (BL41, Blue1, CA46, Ramos, Raji) are illustrated for the 5′- ends of the MS4A1 gene by using the UCSC genome browser (http://genome.ucsc.edu/). Reads in red map to the forward strand and blue reads to the reverse strand. The location of real-time DNA-PCR (Table S1) is schematically indicated above the gene annotations as well as the genomic intervals identified by bioinformatic analysis (Table S5). (PDF) [file pone.0026837.s005.pdf]

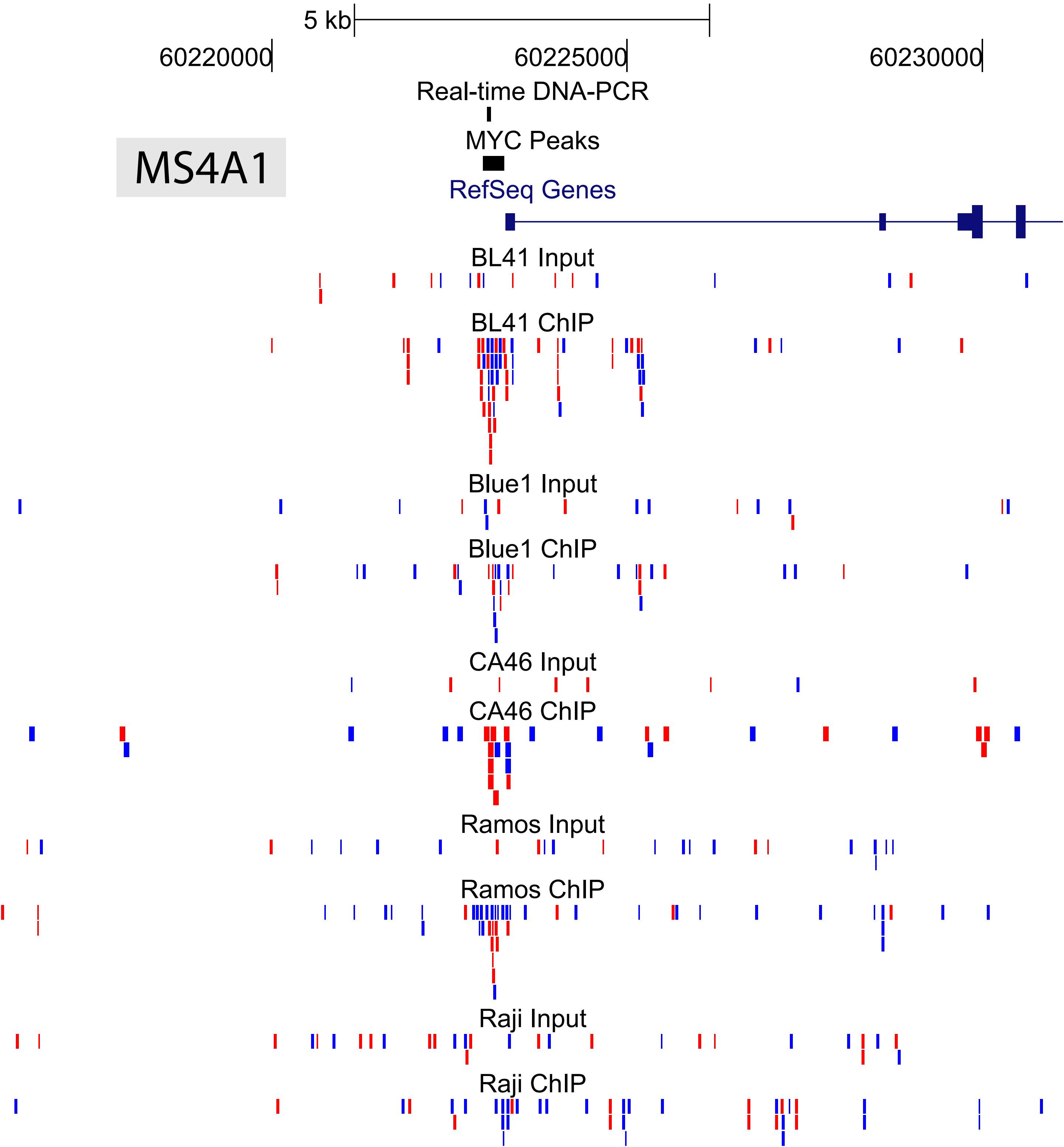

5 kb
60220000
60225000
60230000
Real-time DNA-PCR
MYC Peaks
MS4A1
RefSeq Genes
BL41 Input
BL41 ChIP
Blue1 Input
Blue1 ChIP
CA46 Input
CA46 ChIP
Ramos Input
Ramos ChIP
Raji Input
Raji ChIP
