# Supplementary material for: Deep Sequencing of MYC DNA-Binding Sites in Burkitt Lymphoma
Source: PLoS One. 2011 Nov 10;6(11):e26837. doi: 10.1371/journal.pone.0026837 (PMC3213110; doi:10.1371/journal.pone.0026837)
Supplement: Table S4 — Number of reads in the Fastq files for all MYC ChIP-Seq and input samples mapped to the human hg19 reference genome. The number of reads within the putative peaks discovered by HOMER and within the final list of 7,054 merged peaks (DESeq) is shown in the far right column. (DOC) [file pone.0026837.s013.doc]

**Table S4:** **Number of reads in the Fastq files for all MYC ChIP-Seq and input samples mapped to the human hg19 reference genome.**

The number of reads within the putative peaks discovered by HOMER and within the final list of 7,054 merged peaks (DEseq) is shown in the far right column.
